# Clinical and inflammatory changes following routine scaling and root planing in periodontitis patients with and without diabetes: a prospective observational cohort study

**DOI:** 10.3389/fendo.2026.1937181

**Published:** 2026-09-24

**Authors:** Li Li, Xue Yang, Xuguang Yang, Xiaofei Sun, Xiaoli Sun, Qijuan Dong, Yong Tian

**Affiliations:** Department of Endocrinology and Metabolism, the Fifth Clinical Medical College of Henan University of Chinese Medicine (Zhengzhou People’s Hospital), Zhengzhou, China

**Keywords:** cytokines, diabetes, gingival crevicular fluid, periodontitis, scaling and root planing, systemic immune-inflammation index

## Abstract

**Objective:**

To prospectively observe changes in periodontal clinical outcomes and local and blood-derived inflammatory markers among patients with periodontitis with and without type 2 diabetes following routine scaling and root planing (SRP).

**Methods:**

This prospective observational cohort study consecutively enrolled patients with periodontitis and classified them according to pre-existing diabetes status. SRP was provided as part of routine clinical care and was not assigned or modified by the investigators; no untreated concurrent comparison group was included. Periodontal clinical parameters, gingival crevicular fluid levels of TNF-α, IL-1β, and IL-10, and peripheral blood-derived NLR and SII were assessed at baseline and approximately 3 months. HbA1c was additionally evaluated as an exploratory outcome in participants with diabetes.

**Results:**

At 3 months, PD, BI, and PLI were significantly lower than baseline in both groups (*P* < 0.05), whereas CAL did not change significantly. BI remained higher in the diabetes-associated periodontitis group than in the non-diabetic periodontitis group (*P* = 0.039). Gingival crevicular fluid TNF-α decreased significantly in both groups (*P* < 0.001) but remained higher in the diabetes-associated periodontitis group at 3 months (*P* < 0.001). IL-1β did not change significantly in either group. IL-10 increased significantly in the non-diabetic periodontitis group (*P* < 0.05), whereas the increase in the diabetes-associated periodontitis group was not significant. NLR and SII decreased significantly in both groups, with no significant between-group differences at baseline, 3 months, or in the magnitude of change. In participants with diabetes, HbA1c showed a non-significant numerical decrease from 8.37 ± 1.03% to 8.07 ± 1.06% (*P* = 0.081).

**Conclusion:**

Among patients receiving routine SRP, periodontal clinical parameters and selected inflammatory markers improved over the 3-month follow-up in both groups. Participants with diabetes had higher residual local inflammation at 3 months, reflected by higher BI and gingival crevicular fluid TNF-α levels, whereas no significant between-group differences in changes in blood-derived inflammatory indices were detected. HbA1c decreased numerically but not significantly; therefore, no conclusion regarding improved glycemic control can be drawn.

## 41 Introduction

Periodontitis is a chronic infectious disease initiated by dental plaque biofilm and characterized by progressive destruction of the tooth-supporting tissues. It is one of the main causes of tooth loss in adults. According to data from the Global Burden of Disease Study, severe periodontitis affects about 11% of the world’s population. It remains one of the most prevalent oral diseases and has been recognized by the World Health Organization as an important public health problem (1–3). The pathogenesis of periodontitis involves dysregulation of the host immune-inflammatory response. Periodontal pathogens not only directly invade periodontal tissues but also stimulate the host to release large amounts of pro-inflammatory cytokines, leading to alveolar bone resorption and connective tissue degradation, and ultimately resulting in irreversible periodontal attachment loss (4). In clinical practice, PD, CAL, PLI, and BI are the core clinical parameters used to assess disease severity and activity, and they are also important indicators for evaluating the efficacy of periodontal therapy (5).

Diabetes and periodontitis are closely and bidirectionally associated. Each act as a risk factor for the other and may accelerate disease progression. On the one hand, in patients with diabetes, persistent hyperglycemia impairs immune function, reduces neutrophil chemotaxis and phagocytosis, promotes the accumulation of advanced glycation end products (AGEs), and contributes to microvascular damage. As a result, periodontal tissues become more susceptible to pathogenic bacteria, and both the prevalence and severity of periodontitis are higher than in non-diabetic populations (6). On the other hand, chronic periodontal infection may aggravate systemic low-grade inflammation through the release of large amounts of pro-inflammatory mediators, thereby promoting insulin resistance and disturbing glycemic homeostasis, which in turn leads to elevated glycated hemoglobin (HbA1c) levels (7). At present, HbA1c is widely used to monitor long-term glycemic control in patients with diabetes, and longitudinal changes in HbA1c are clinically relevant when characterizing metabolic patterns following periodontal treatment. Previous systematic reviews and meta-analyses have reported modest average reductions in HbA1c following periodontal therapy in patients with type 2 diabetes, further supporting the close relationship between periodontal inflammation and glycemic status (8–10). Therefore, under diabetic conditions, it is of considerable theoretical and clinical importance to characterize the clinical, inflammatory, and metabolic changes observed following routine periodontal treatment.

Gingival crevicular fluid (GCF), as a direct reflection of the local periodontal microenvironment, contains a wide range of biomarkers closely related to periodontal inflammation and tissue destruction, and has become an important window for monitoring local inflammatory status in periodontal disease. Among these markers, TNF-α is a key pro-inflammatory cytokine secreted by macrophages and gingival fibroblasts. It can induce matrix metalloproteinase expression and activate osteoclasts, thereby playing a critical role in alveolar bone resorption. IL-1β is another major driver of the local inflammatory response in periodontitis. Its concentration in GCF is significantly positively correlated with clinical parameters such as PD and CAL, and it is regarded as a sensitive marker of ongoing periodontal tissue destruction (11, 12). In contrast to these pro-inflammatory mediators, IL-10 is an important anti-inflammatory cytokine that suppresses the production of pro-inflammatory factors and helps maintain immune homeostasis. Changes in its level within the periodontal inflammatory microenvironment reflect the dynamic balance between pro-inflammatory and anti-inflammatory responses. In periodontitis patients with diabetes, the expression levels of TNF-α and IL-1β in GCF are often higher, whereas the regulatory capacity of IL-10 is relatively impaired. This suggests that diabetes may aggravate periodontal tissue destruction by altering the local immune-inflammatory response (13, 14). Therefore, monitoring dynamic changes in these inflammatory mediators in GCF may provide a more comprehensive cell- and molecule-based evaluation of the local anti-inflammatory effects of periodontal therapy.

In recent years, systemic inflammatory markers derived from routine blood cell counts have received increasing attention. Among them, the NLR and the SII are easy to obtain and relatively inexpensive, and they have been widely used for risk assessment and disease monitoring in cardiovascular disease, metabolic syndrome, and various chronic inflammatory disorders. NLR mainly reflects the balance between neutrophil-mediated inflammatory activation and lymphocyte-mediated immune regulation. SII further integrates three parameters—neutrophils, lymphocytes, and platelets—and therefore provides a more comprehensive measure of systemic immune-inflammatory status (15, 16). Although periodontitis presents clinically as a localized infectious disease of the oral cavity, it is closely associated with chronic low-grade systemic inflammation. Periodontal pathogens and their metabolic products may enter the bloodstream and trigger host immune-inflammatory responses, thereby increasing NLR and SII (17). In patients with diabetes, these inflammatory markers are usually higher than in healthy individuals because of the underlying chronic inflammatory state and immune dysregulation. When periodontitis is also present, the overall inflammatory burden may be further increased.

SRP is the core procedure in non-surgical periodontal therapy. By mechanically removing supragingival and subgingival plaque, calculus, and contaminated root surfaces, SRP reduces the periodontal pathogen burden and local inflammatory stimulation. A recent study by Yue et al. evaluated periodontal clinical parameters and gingival crevicular fluid TNF-α, IL-1β, and IL-10 before and 3 months after SRP in 76 patients with periodontitis with or without diabetes (18). However, that study did not assess peripheral blood-cell components or composite blood-derived inflammatory indices. Whether local periodontal changes are accompanied by measurable changes in NLR and SII therefore remains insufficiently characterized. The present study addressed this question by integrating periodontal clinical outcomes, gingival crevicular fluid cytokines, platelet, neutrophil, and lymphocyte counts, and the derived NLR and SII (19, 20).

Therefore, this prospective observational study aimed to characterize changes in periodontal clinical outcomes and local and blood-derived inflammatory markers between the routine pretreatment assessment and the 3-month clinical follow-up among patients with periodontitis with and without type 2 diabetes. We further explored whether the observed longitudinal patterns differed according to pre-existing diabetes status. HbA1c was evaluated as an exploratory metabolic measure in participants with diabetes.

## 2 Materials and methods

### 2.1 Study design and ethical statement

This was a prospective observational cohort study with repeated measurements. Patients with periodontitis were consecutively enrolled and categorized according to their pre-existing diabetes status into a diabetes-associated periodontitis group and a non-diabetic periodontitis group. Diabetes status was not assigned or modified by the investigators. All participants were patients for whom scaling and root planing had been clinically indicated and provided as part of routine periodontal care. The clinical indication for periodontal treatment was determined by the treating periodontist independently of study enrollment. Once SRP was clinically indicated, treatment was delivered according to the hospital’s routine periodontal care protocol. The study did not assign participants to an intervention, withhold treatment, or modify routine clinical management. Clinical periodontal findings and laboratory measurements obtained at the routine pretreatment visit and approximately 3 months later were prospectively recorded for observational comparison. All study procedures were conducted in accordance with the ethical principles of the Declaration of Helsinki. The study protocol was approved by the Medical Ethics Committee of Zhengzhou People’s Hospital (Approval No. KY-2024-002-01). Before enrollment, all participants were fully informed of the observational data and sample collection procedures and provided with written informed consent.

### 2.2 Study population

From June 2025 to March 2026, patients attending Zhengzhou People’s Hospital with clinically diagnosed Stage II–III periodontitis who underwent scaling and root planing for routine clinical indications were screened for eligibility in chronological order according to their presentation during the recruitment period. Eligible patients were consecutively invited to participate without selective sampling. Clinical and laboratory information was prospectively collected at the pretreatment visit and at the routine follow-up visit approximately 3 months later. According to diabetes status, the participants were divided into two groups: the diabetes-associated periodontitis group, which included patients who met both the World Health Organization diagnostic criteria for diabetes (HbA1c ≥ 6.5% or fasting blood glucose ≥ 7.0 mmol/L) and the diagnostic criteria for periodontitis; and the non-diabetic periodontitis group, which included patients with periodontitis but without a history of diabetes or related metabolic abnormalities (21). Eligibility for the study did not determine whether a patient received scaling and root planing, and patients who declined research participation continued to receive the same routine clinical care.

Periodontitis was diagnosed and staged according to the 2017 World Workshop classification of periodontal and peri-implant diseases and conditions. A patient was considered to have periodontitis when interdental clinical attachment loss (CAL) was detectable at ≥2 non-adjacent teeth, or when buccal/oral CAL ≥3 mm with periodontal pocketing >3 mm was present at ≥2 teeth, provided that the attachment loss could not be attributed to non-periodontitis-related causes. Staging was based on disease severity and complexity, with interdental CAL used as the primary determinant. Stage II periodontitis was defined principally by interdental CAL of 3–4 mm and a maximum probing depth of ≤5 mm, without complexity features requiring classification as a higher stage. Stage III periodontitis was defined principally by interdental CAL ≥5 mm or by the presence of Stage III complexity features, such as probing depth ≥6 mm, vertical bone loss ≥3 mm, class II or III furcation involvement, or limited tooth loss attributable to periodontitis. When severity and complexity criteria indicated different stages, the higher stage was assigned. Only patients classified as Stage II or Stage III at the pretreatment assessment were eligible for the present study (22, 23).

The inclusion criteria were as follows: (1) diagnosis of Stage II or Stage III periodontitis according to the 2017 World Workshop classification; (2) age between 18 and 65 years; (3) at least 20 natural teeth remaining, with no fewer than 5 teeth in each quadrant; and (4) no systemic antibiotic therapy or professional periodontal treatment within the previous 6 months.

The exclusion criteria were as follows: (1) presence of other active systemic inflammatory diseases or autoimmune disorders; (2) pregnancy or lactation; (3) continuous use of immunosuppressants, non-steroidal anti-inflammatory drugs, or glucocorticoids within the previous 3 months; (4) a history of systemic hormone therapy, radiotherapy, or chemotherapy within the previous 6 months.

Participant-reported information collected using the study-specific questionnaire was reviewed by a trained investigator and, where applicable, verified against medical records and clinical examination findings.

### 2.3 Sample size calculation

The sample size was calculated *a priori* for the primary between-group comparison of gingival crevicular fluid inflammatory responses. A moderate-to-large standardized mean difference (Cohen’s d = 0.69) was prespecified as the minimum effect of interest. Using G*Power for a two-sided independent-samples comparison with α = 0.05, 80% power, and an anticipated group-size ratio of approximately 1:1, the minimum required sample size was 34 participants per group. After allowing for approximately 10% attrition or sample exclusion, the target sample size was increased to at least 38 participants in each naturally occurring diabetes-status group. Ultimately, 96 participants completed both baseline and 3-month assessments and were included in the longitudinal analysis, with 48 participants in each group. The calculation was based on the principal inflammatory comparison; HbA1c and the peripheral blood-derived indices were treated as secondary or exploratory outcomes.

### 2.4 Baseline data collection and clinical assessment

After enrollment, all participants completed a study-specific structured data-collection questionnaire and underwent a clinical examination. The questionnaire was developed by investigators specifically for the present study based on the prespecified study design, eligibility criteria, and variables required for the planned analyses; it has not been previously published elsewhere. The questionnaire was used to collect demographic characteristics, anthropometric information, smoking and alcohol consumption, relevant medical history, medication use, diabetes-related information, and information required to assess study eligibility. It was not designed as a psychometric or diagnostic scale, did not generate a composite score, and was not used to measure the primary or secondary study outcomes. General baseline variables included age, sex, height, weight, body mass index (BMI), smoking status, and alcohol consumption. For participants in the diabetes-associated periodontitis group, diabetes-related information included diabetes type and duration, glucose-lowering treatment regimen, fasting blood glucose, HbA1c, and diabetes-related complications. Baseline medication use was recorded for all participants, and the glucose-lowering treatment regimen was additionally documented for participants with diabetes. However, changes in glucose-lowering therapy or other medications during the approximately 3-month observation period were not systematically captured as standardized longitudinal study variables. An English-language version of the questionnaire is provided as **Supplementary Material 1**. Questionnaire items related to oral status and final eligibility were completed or confirmed by the investigator rather than self-reported by the participants.

All periodontal examinations were performed by the same examiner, who had undergone calibration training and achieved good intra-examiner agreement (Kappa > 0.85). The pretreatment periodontal examination was also used to confirm the periodontitis diagnosis and stage for study eligibility. Staging was determined at the patient level according to the most severe qualifying site and the presence of relevant complexity features, rather than from the mean full-mouth PD or CAL values used in the longitudinal analyses. A Williams periodontal probe was used with a standardized probing force of approximately 20 g. The same clinical and laboratory outcomes were measured in each participant at baseline and at the 3-month follow-up, allowing assessment of within-participant changes over time and between-group differences according to diabetes status. The periodontal clinical parameters recorded at baseline and at the 3-month follow-up included probing depth (PD), clinical attachment level (CAL), bleeding index (BI), and plaque index (PLI). PD was defined as the distance from the gingival margin to the bottom of the periodontal pocket and was recorded in millimeters using a Williams periodontal probe (22, 23). CAL was defined as the distance from the cementoenamel junction to the bottom of the periodontal pocket and was also recorded in millimeters (22, 23). Gingival bleeding was assessed using the bleeding index (BI) (24), with bleeding severity evaluated approximately 30 seconds after probing on a 0–5 ordinal scale: 0, clinically healthy gingiva without bleeding; 1, inflammatory color change without bleeding on probing; 2, slight point bleeding; 3, bleeding extending along the gingival margin; 4, profuse bleeding filling or overflowing the gingival sulcus; and 5, spontaneous bleeding. Higher BI scores therefore indicated greater gingival bleeding severity. Plaque accumulation was assessed using the Silness–Löe plaque index (PLI) on a 0–3 scale (25), where 0 indicated no plaque, 1 a thin plaque film detectable by probing, 2 moderate visible plaque accumulation, and 3 abundant plaque accumulation. The same periodontal examination procedures were applied at baseline and at the 3-month follow-up.

Following the routine pretreatment assessment, participants underwent full-mouth subgingival SRP as part of standard periodontal care. SRP was completed in two treatment sessions within one week, with the left and right sides of the dentition treated separately. The interval between the two sessions was 3–6 days. Treatment was performed by the same experienced periodontist under local anesthesia using ultrasonic scalers and hand instruments according to the hospital’s standardized clinical practice. All participants received oral hygiene instruction, including the modified Bass toothbrushing technique and guidance on the appropriate use of interdental cleaning devices. Adjunctive antibiotic therapy was not routinely required by the study protocol, and any antibiotic exposure during follow-up was recorded where applicable. Supportive periodontal care was scheduled at an approximately 3-month recall interval. At the 3-month visit, periodontal examination and study-related sample collection were completed before any additional periodontal instrumentation. Thereafter, sites with residual probing depth (PD) ≥4 mm underwent additional subgingival instrumentation as clinically indicated.

### 2.5 Collection of gingival crevicular fluid and measurement of inflammatory mediators

At baseline, two non-adjacent diseased teeth within the same quadrant were selected as participant-specific GCF sampling teeth. Candidate sampling teeth with obvious hard-tissue defects, wedge-shaped defects, furcation involvement, or extensive restorations that could compromise sample quality were not selected as GCF sampling sites. Two non-adjacent diseased sites were used to obtain a participant-level representation of the local periodontal inflammatory environment while avoiding reliance on a single periodontal site. These teeth were designated as fixed sampling teeth and were intended to be resampled at the 3-month follow-up so that longitudinal comparisons were based, whenever possible, on the same anatomical sites. If a prespecified target tooth was no longer suitable for sampling because it was missing or severely decayed, the corresponding tooth in an adjacent quadrant was selected as a replacement according to the same clinical sampling principles. Before sampling, the area was isolated with cotton rolls and gently air-dried to minimize saliva contamination. A standardized paper point was inserted approximately 1 mm below the entrance of the periodontal pocket and maintained in position for 30 seconds. Samples with visible blood contamination were discarded, and sampling was repeated at an alternative eligible diseased site. The two GCF samples obtained from each participant at each assessment were pooled into a single pre-labeled microcentrifuge tube containing 200 μL of sterile phosphate-buffered saline (PBS) and were subsequently analyzed as one participant-level sample. Pooling was performed to generate a participant-level GCF measurement for the planned longitudinal analyses; consequently, cytokine concentrations from the two individual periodontal sites were not retained or analyzed separately. The samples were immediately stored in an ice box and transferred to a −80 °C freezer within 4 hours until analysis.

Before analysis, the GCF samples were slowly thawed at room temperature and vortex-mixed for 30 seconds to ensure homogenization. They were then centrifuged at 12,000 × *g* for 15 minutes at 4 °C, and the supernatants were collected for further analysis. Commercial enzyme-linked immunosorbent assay (ELISA) kits were used to quantify the concentrations of TNF-α, IL-1β, and IL-10 according to the manufacturers’ instructions. For each assay plate, a standard curve with eight concentration gradients and quality-control samples at high, medium, and low concentrations were included. All samples and standards were tested in duplicate, and the mean values were used for analysis. Absorbance was measured at 450 nm using a microplate reader (Multiskan FC, Thermo Scientific, USA). The results were expressed in pg/mL.

### 2.6 Collection and calculation of systemic inflammatory indices

Available complete blood count results obtained during routine clinical assessments at baseline and approximately 3 months were prospectively recorded. Complete blood counts were performed in the same hospital laboratory using a standardized automated hematology analyzer. The participant-level platelet count and absolute neutrophil and lymphocyte counts were extracted at both assessment time points. The composite systemic inflammatory indices were calculated using the following formulas:

Neutrophil-to-lymphocyte ratio (NLR):


$$NLR=\frac{\text{neutrophil count }(\times 10^{9}/\text{L})}{\text{lymphocyte count }(\times 10^{9}/\text{L})}$$

Systemic immune-inflammation index (SII):


$$SII=\frac{\text{platelet count }(\times 10^{9}/\text{L})\times \text{neutrophil count }(\times 10^{9}/\text{L})}{\text{lymphocyte count }(\times 10^{9}/\text{L})}$$

NLR was calculated as the absolute neutrophil count divided by the absolute lymphocyte count, and SII was calculated as the platelet count multiplied by the absolute neutrophil count and divided by the absolute lymphocyte count (26, 27). HbA1c was additionally measured at baseline and at the 3-month follow-up in participants in the diabetes-associated periodontitis group and was analyzed as an exploratory metabolic outcome.

### 2.7 Statistical analysis

All statistical analyses were performed using SPSS version 27.0 (IBM Corp., Armonk, NY, USA). The longitudinal analyses used a complete-case approach and included participants who completed both the baseline and approximately 3-month assessments; no imputation of missing follow-up data was performed. Continuous variables were assessed for normality using the Shapiro–Wilk test together with visual inspection of Q–Q plots. Normally distributed continuous variables are presented as mean ± standard deviation, non-normally distributed variables as median (interquartile range), and categorical variables as frequencies and percentages.

Given the two-time-point repeated-measures design, within-participant changes from baseline to 3 months were assessed using paired-samples t tests for approximately normally distributed paired differences or Wilcoxon signed-rank tests for non-normally distributed paired data. To evaluate whether the magnitude of longitudinal change differed between participants with and without diabetes, participant-level change values were calculated as the baseline value minus the corresponding 3-month value and compared between groups using independent-samples t tests or Mann–Whitney U tests, as appropriate. Between-group comparisons at baseline and at 3 months were performed using independent-samples t tests or Mann–Whitney U tests according to data distribution. Categorical variables were compared using the chi-square test or Fisher’s exact test, as appropriate. Attrition proportions between the diabetes and non-diabetes groups were compared using Fisher’s exact test.

Platelet count, absolute neutrophil count, absolute lymphocyte count, neutrophil-to-lymphocyte ratio (NLR), and systemic immune-inflammation index (SII) were non-normally distributed in at least one study group or assessment time point and were therefore summarized as median (interquartile range) and analyzed using non-parametric tests. Gingival crevicular fluid cytokine concentrations were analyzed at the participant level because samples obtained from the two selected periodontal sites were pooled before measurement. HbA1c was assessed only in participants with diabetes and was treated as an exploratory metabolic outcome. Because the paired HbA1c differences were approximately normally distributed, baseline and 3-month values were compared using the paired-samples t test, with the mean change and 95% confidence interval reported.

The primary analyses were unadjusted. Although BMI differed significantly between groups at baseline, it was not included as an adjustment covariate. All participants received the same routine SRP schedule, oral hygiene instruction, and approximately 3-month supportive periodontal recall; however, individual adherence to home oral hygiene recommendations and longitudinal changes in medication use or diabetes management were not systematically quantified as standardized covariates and were therefore not included in adjusted analyses. The present analytical approach accounted for within-participant pairing but did not model group and time simultaneously within a formal repeated-measures or mixed-effects framework. Accordingly, between-group *P* values for change represent comparisons of participant-level longitudinal changes and should not be interpreted as formal group × time interaction tests. All statistical tests were two-sided, and *P* < 0.05 was considered statistically significant.

## 3 Results

### 3.1 Baseline demographic characteristics

A total of 122 patients were assessed for eligibility at baseline. Fourteen were excluded during screening, and 108 eligible participants entered prospective observational follow-up, including 52 participants with diabetes-associated periodontitis and 56 with non-diabetic periodontitis. Twelve participants (11.1%) did not complete the approximately 3-month reassessment: five had relocated away from the study area and were unable to attend the follow-up visit, whereas seven declined further participation because of time constraints. Of the 12 participants who lost to follow-up, 4 were in the diabetes group and 8 were in the non-diabetes group, corresponding to attrition rates of 7.7% and 14.3%, respectively. The difference in attrition between groups was not statistically significant. Ultimately, 96 participants completed both the baseline and 3-month assessments and were included in the complete-case longitudinal analysis, with 48 participants in each group (**Figure 1**).

**Figure 1:**
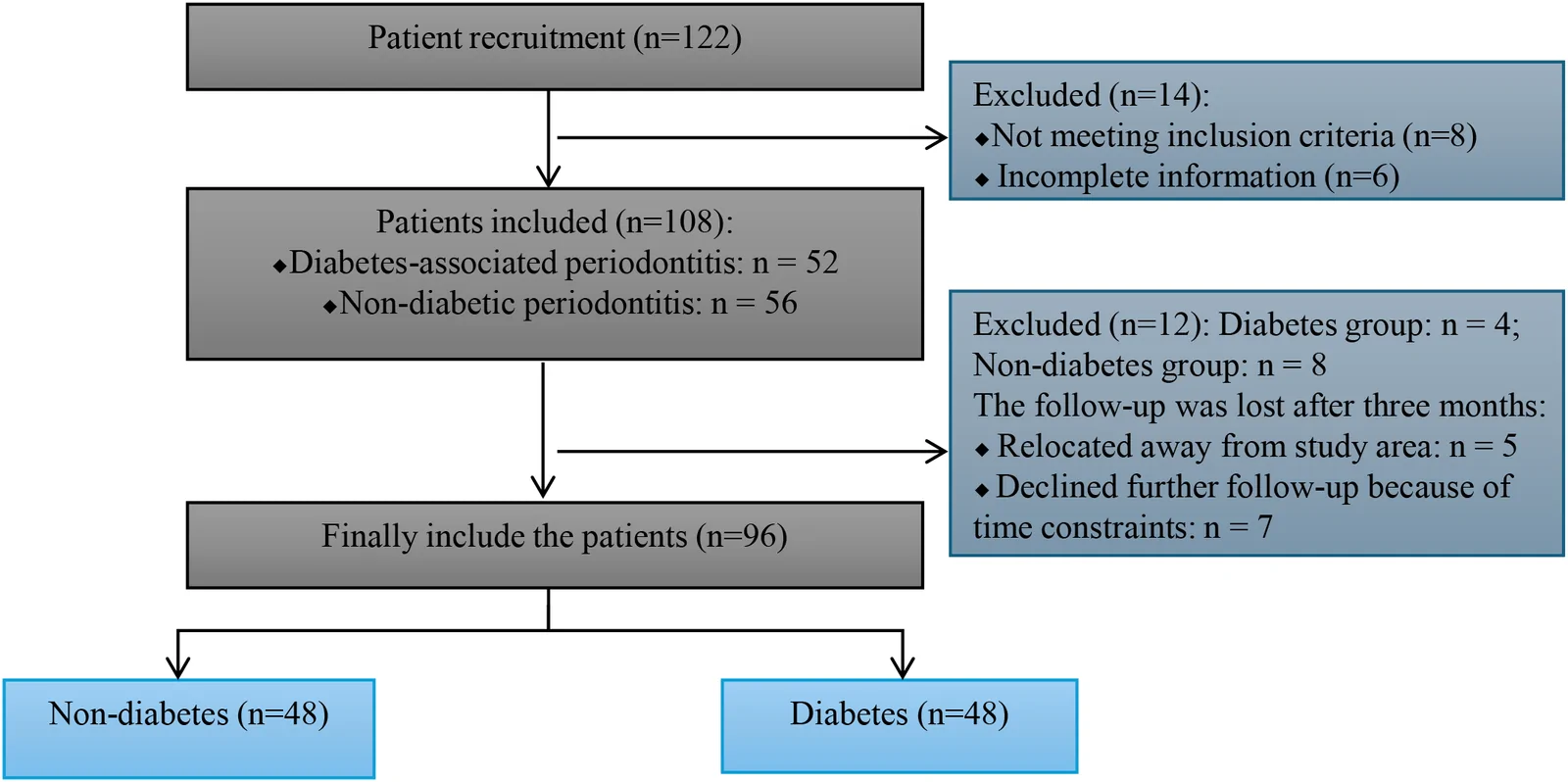
Flow diagram of participant recruitment and follow-up.

The baseline demographic characteristics and periodontal clinical parameters of the two groups are shown in **Table 1**. The groups were generally comparable in age, sex, smoking status, and alcohol consumption. Mean age was 45.63 ± 7.71 years in the diabetes group and 45.25 ± 6.02 years in the non-diabetic group (*P* = 0.791). The non-diabetic group included 24 men and 24 women, and the diabetes group included 26 men and 22 women (*P* = 0.683). Smoking and alcohol consumption also did not differ significantly between groups (*P* = 0.537 and *P* = 0.072, respectively).

**Table 1:** Demographic characteristics and baseline periodontal parameters of the 96 participants.

Participants’ demographics (n = 96)	Non-diabetic (n = 48)	Diabetes (n=48)	*P* value
Age (years)	45.25 ± 6.02	45.63 ± 7.71	0.791
Sex			0.683
Male	24	26	
Female	24	22	
BMI			0.003
Underweight/normal	45	34	
Overweight/obesity	3	14	
Smoking			0.537
No	41	43	
Yes	7	5	
Alcohol consumption			0.072
No	38	30	
Yes	10	18	
Probing depth (PD) (mm)			0.626
Mean ± SD	3.92 ± 0.87	4.00 ± 0.80	
Maximum value	6	6	
Minimum value	2	2	
Clinical attachment level (CAL) (mm)			0.397
Mean ± SD	5.56 ± 1.37	5.79 ± 1.27	
Maximum value	9	9	
Minimum value	2	3	
Bleeding index (BI)			0.603
Mean ± SD	2.19 ± 0.61	2.25 ± 0.57	
Maximum value	4	3	
Minimum value	1	1	
Plaque index (PLI)			0.657
Mean ± SD	1.94 ± 0.52	1.98 ± 0.39	
Maximum value	3	3	
Minimum value	1	1	

Both groups consisted of patients with periodontitis. Non-diabetic, non-diabetic periodontitis group; Diabetes, diabetes-associated periodontitis group. BMI, Body Mass Index; SD, Standard Deviation.

Notably, BMI distribution differed significantly between the two groups at baseline (*P* = 0.003). Overweight or obesity was present in 14 of 48 participants (29.2%) in the diabetes-associated periodontitis group compared with 3 of 48 participants (6.3%) in the non-diabetic periodontitis group, whereas 34 (70.8%) and 45 (93.8%) participants, respectively, were classified as underweight or normal weight. Thus, BMI represented an important baseline imbalance between the groups and was considered when interpreting subsequent between-group comparisons.

Baseline periodontal clinical parameters did not differ significantly between groups. PD was 3.92 ± 0.87 mm in the non-diabetic group and 4.00 ± 0.80 mm in the diabetes group (*P* = 0.626); CAL was 5.56 ± 1.37 mm and 5.79 ± 1.27 mm, respectively (*P* = 0.397); BI was 2.19 ± 0.61 and 2.25 ± 0.57 (*P* = 0.603); and PLI was 1.94 ± 0.52 and 1.98 ± 0.39 (*P* = 0.657).

### 3.2 Changes in clinical parameters

Three months after SRP, the periodontal clinical parameters in both groups showed an overall trend toward improvement. The detailed results are presented in **Table 2** and **Figure 2**. In general, both the non-diabetes group and the diabetes group showed clear improvements in PD, BI, and PLI compared with baseline, whereas the improvement in CAL was relatively limited and did not reach statistical significance.

**Table 2:** Periodontal clinical parameters and HbA1c at baseline and 3 months.

Periodontal parameters (mean ± SD)	Evaluation time point		Difference
	Baseline	3 months	BL-3 months
PD (mm)	3.96 ± 0.83	3.28 ± 0.59	0.68 ± 1.02
Non-diabetic	3.92 ± 0.87	3.19 ± 0.49	0.73 ± 1.05
Diabetes	4.00 ± 0.80	3.38 ± 0.67	0.63 ± 1.00
*P* value	0.626	0.122	0.620
CAL (mm)	5.68 ± 1.32	5.23 ± 1.13	0.45 ± 1.73
Non-diabetic	5.56 ± 1.37	5.17 ± 0.91	0.40 ± 1.54
Diabetes	5.79 ± 1.27	5.29 ± 1.32	0.50 ± 1.91
*P* value	0.397	0.590	0.948
BI	2.22 ± 0.58	1.78 ± 0.55	0.44 ± 0.78
Non-diabetic	2.19 ± 0.61	1.67 ± 0.56	0.52 ± 0.74
Diabetes	2.25 ± 0.57	1.90 ± 0.52	0.35 ± 0.81
*P* value	0.692	0.039	0.297
PLI	1.96 ± 0.46	1.60 ± 0.55	0.35 ± 0.68
Non-diabetic	1.94 ± 0.52	1.58 ± 0.54	0.35 ± 0.67
Diabetes	1.98 ± 0.39	1.63 ± 0.57	0.35 ± 0.70
*P* value	0.657	0.714	0.999
HbA1c (%)	8.37 ± 1.03	8.07 ± 1.06	0.30 ± 1.17

Both groups consisted of patients with periodontitis. Non-diabetic, non-diabetic periodontitis group; Diabetes, diabetes-associated periodontitis group. BL, Baseline; PD, probing depth; CAL, clinical attachment level; BI, bleeding index (0–5 scale; higher scores indicate greater bleeding severity); PLI, plaque index (0–3 scale; higher scores indicate greater plaque accumulation); SRP, scaling and root planing.

**Figure 2:**
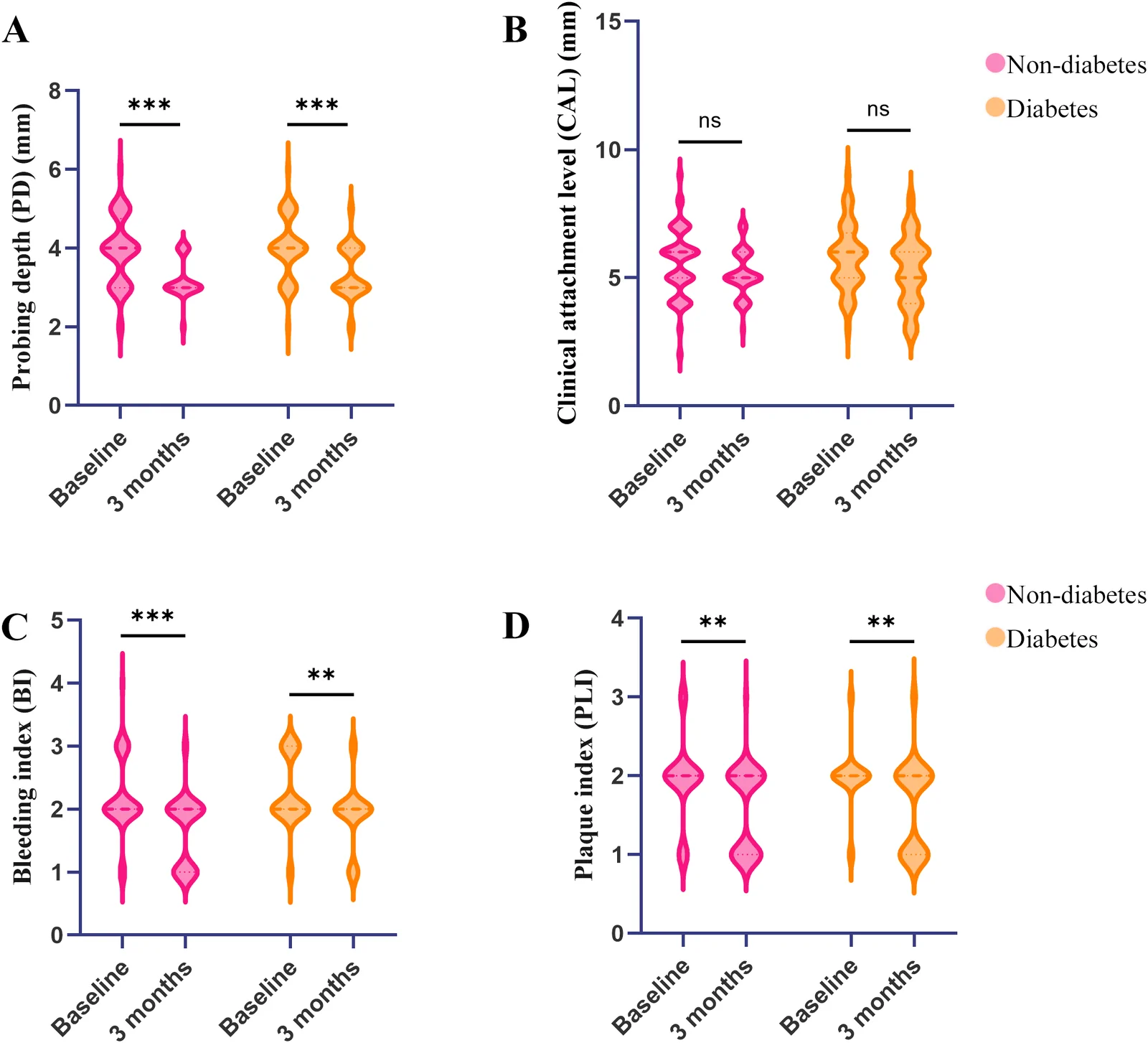
Changes in periodontal clinical parameters from baseline to 3 months after scaling and root planing. **(A)** Probing depth; **(B)** clinical attachment level; **(C)** bleeding index; and **(D)** plaque index. PD, probing depth; CAL, clinical attachment level; BI, bleeding index; PLI, plaque index. ** P<0.01, *** P<0.001; ns indicates no statistically significant difference.

For PD, both groups showed a significant reduction after treatment. In the non-diabetes group, PD decreased from 3.92 ± 0.87 mm to 3.19 ± 0.49 mm, while in the diabetes group it decreased from 4.00 ± 0.80 mm to 3.38 ± 0.67 mm. **Figure 2A** also shows a significant difference between baseline and 3 months after treatment in both groups (*P* < 0.001). Further between-group comparisons showed no significant differences in baseline values, 3-month values, or change values (*P* > 0.05), indicating that the magnitude of the 3-month change in PD did not differ significantly according to diabetes status.

For CAL, both groups showed a downward trend after treatment, but the within-group changes did not reach statistical significance. In the non-diabetes group, CAL decreased from 5.56 ± 1.37 mm to 5.17 ± 0.91 mm, while in the diabetes group it decreased from 5.79 ± 1.27 mm to 5.29 ± 1.32 mm. **Figure 2B** similarly shows that the differences between baseline and 3 months after treatment were not statistically significant in either group (*P* > 0.05). In addition, no significant between-group differences were found in baseline values, post-treatment values, or change values (*P* > 0.05). These findings indicate that CAL changed little over the 3-month period following routine SRP and that the pattern of change was similar in the two groups.

For BI, both groups showed a marked decrease after treatment. In the non-diabetes group, BI decreased from 2.19 ± 0.61 to 1.67 ± 0.56, and this reduction was statistically significant (*P* < 0.001). In the diabetes group, BI decreased from 2.25 ± 0.57 to 1.90 ± 0.52, which was also statistically significant (*P* < 0.01) (**Figure 2C**). Notably, the between-group difference in BI at 3 months after treatment was statistically significant (*P* = 0.039), with the non-diabetes group showing a lower BI than the diabetes group. However, the difference in the magnitude of BI change between the two groups was not statistically significant (*P* = 0.297). This suggests that although the diabetes group had a relatively higher level of residual inflammation after treatment, the overall degree of improvement was similar in the two groups.

For PLI, both groups showed a significant reduction after treatment. In the non-diabetes group, PLI decreased from 1.94 ± 0.52 to 1.58 ± 0.54, while in the diabetes group it decreased from 1.98 ± 0.39 to 1.63 ± 0.57. In both groups, the within-group changes were statistically significant (*P* < 0.01) (**Figure 2D**). Between-group comparisons showed no significant differences in baseline values, 3-month values, or change values (*P* > 0.05), indicating that the pattern of change in PLI over the 3-month follow-up was broadly similar in the two groups.

In the diabetes-associated periodontitis group, mean HbA1c changed numerically from 8.37 ± 1.03% at baseline to 8.07 ± 1.06% at 3 months, corresponding to a numerical decrease of 0.30 percentage points. However, the paired difference was not statistically significant (P = 0.081). Thus, the HbA1c findings do not provide evidence of improved glycemic control and should be regarded as exploratory (**Table 2**; **Supplementary Figure S1**).

At 3 months after SRP, PD, BI, and PLI were lower than at baseline in both groups, whereas CAL did not change significantly. Except for the higher BI in the diabetes group at 3 months, between-group differences in clinical change values were not statistically significant.

### 3.3 Changes in gingival crevicular fluid inflammatory mediators

Three months after SRP, the gingival crevicular fluid inflammatory mediators showed different patterns of change in the two groups. The detailed results are presented in **Table 3** and **Figure 3**.

**Table 3:** Changes in gingival crevicular fluid inflammatory mediator levels from baseline to 3 months after scaling and root planing.

Inflammatory mediator	Group	Baseline	3 months	Baseline − 3 months	Between-group P value at baseline	Between-group P value at 3 months	Between-group P value for change
TNF-α (pg/mL)	Non-diabetic	1278.88 ± 425.87	693.40 ± 146.41	585.48 ± 446.40	0.763	<0.001	0.233
	Diabetes	1302.33 ± 327.10	819.23 ± 134.46	483.10 ± 387.28			
IL-1β (pg/mL)	Non-diabetic	3288.98 ± 886.98	3012.50 ± 600.10	276.48 ± 1122.21	0.857	0.448	0.775
	Diabetes	3318.29 ± 686.16	3101.56 ± 543.73	216.73 ± 913.81			
IL-10 (pg/mL)	Non-diabetic	13.21 ± 2.91	14.81 ± 3.44	−1.60 ± 4.64	0.814	0.308	0.346
	Diabetes	13.33 ± 2.27	14.08 ± 3.51	−0.75 ± 4.16			

Both groups consisted of patients with periodontitis. Non-diabetic, non-diabetic periodontitis group; Diabetes, diabetes-associated periodontitis group. Data are presented as mean ± standard deviation. Change was calculated as the baseline value minus the 3-month value; therefore, a negative value indicates an increase after treatment. Between-group comparisons were performed using the independent-samples t test. Within-group comparisons between baseline and 3 months were performed using the paired-samples t test. TNF-α, tumor necrosis factor alpha; IL, interleukin.

**Figure 3:**
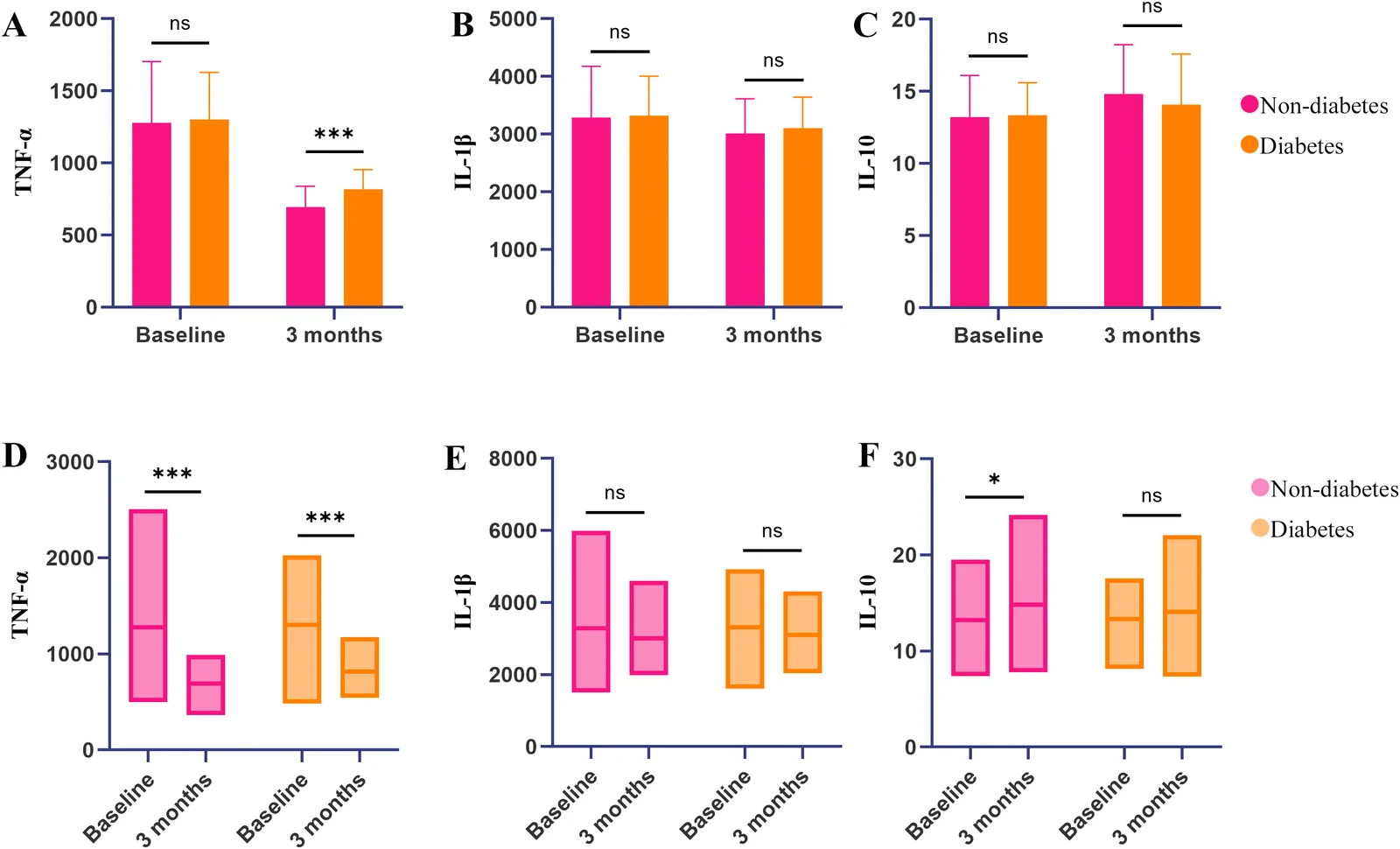
Changes in gingival crevicular flu id cytokine levels after scaling and root planing. **(A–C)** Bar graphs showing bet ween-group comparisons of TNF-α **(A)**, IL-1β **(B)**, and IL-10 **(C)** at baseline and 3 months. **(D–F)** Box plots showing with in-group changes in TNF-α **(D)**, IL-1β **(E)**, and IL-10 **(F)** from baseline to 3 months. *P < 0.05, **P < 0.01, ***P < 0.001; ns, not significant.

For TNF-α, there was no significant difference between the two groups at baseline (*P* = 0.763). Three months after treatment, the TNF-α level was significantly lower in the non-diabetes group than in the diabetes group (*P* < 0.001), whereas the between-group difference in the magnitude of change was not statistically significant (*P* = 0.233) (**Figure 3A**). Specifically, in the non-diabetes group, TNF-α decreased from 1278.88 ± 425.87 pg/mL to 693.40 ± 146.41 pg/mL, with a mean reduction of 585.48 ± 446.40 pg/mL. In the diabetes group, it decreased from 1302.33 ± 327.10 pg/mL to 819.23 ± 134.46 pg/mL, with a mean reduction of 483.10 ± 387.28 pg/mL. Within-group comparisons further showed that TNF-α levels were significantly lower at 3 months than at baseline in both groups, and **Figure 3D** also demonstrates significant differences between baseline and post-treatment values in both groups (*P* < 0.001). These findings indicate that SRP significantly reduced TNF-α levels in both groups. Although the post-treatment absolute level was lower in the non-diabetes group, the overall magnitude of improvement was similar between groups.

For IL-1β, no significant between-group differences were found in baseline levels (*P* = 0.857), post-treatment levels at 3 months (*P* = 0.448), or change values (*P* = 0.775) (**Figure 3B**). Specifically, in the non-diabetes group, IL-1β decreased from 3288.98 ± 886.98 pg/mL to 3012.50 ± 600.10 pg/mL, with a mean reduction of 276.48 ± 1122.21 pg/mL. In the diabetes group, it decreased from 3318.29 ± 686.16 pg/mL to 3101.56 ± 543.73 pg/mL, with a mean reduction of 216.73 ± 913.81 pg/mL. Within-group comparisons showed that IL-1β levels decreased slightly at 3 months in both groups, but the changes did not reach statistical significance (*P* > 0.05) (**Figure 3E**). These results indicate that IL-1β changed little over the 3-month period following routine SRP and that the pattern of change did not differ significantly according to diabetes status.

For IL-10, there were no significant between-group differences in baseline levels (*P* = 0.814), post-treatment levels at 3 months (*P* = 0.308), or change values (*P* = 0.346) (**Figure 3C**). In the non-diabetes group, IL-10 increased from 13.21 ± 2.91 pg/mL to 14.81 ± 3.44 pg/mL, with a mean increase of 1.60 ± 4.64 pg/mL. In the diabetes group, it increased from 13.33 ± 2.27 pg/mL to 14.08 ± 3.51 pg/mL, with a mean increase of 0.75 ± 4.16 pg/mL. Further within-group analysis showed that IL-10 was significantly higher at 3 months than at baseline in the non-diabetes group (*P* < 0.05). By contrast, although an increasing trend was also observed in the diabetes group, the change was not statistically significant (*P* > 0.05) (**Figure 3F**). These findings suggest that the increase in the anti-inflammatory mediator IL-10 after SRP was more pronounced in the non-diabetes group. However, from the between-group perspective, neither the overall levels nor the magnitude of change differed significantly between groups.

### 3.4 Changes in peripheral blood-cell components and inflammatory indices

The platelet count and absolute neutrophil and lymphocyte counts used to reconstruct NLR and SII are summarized in **Supplementary Table S1**. The derived indices are presented in **Table 4** and **Figure 4**.

**Table 4:** Changes in peripheral blood inflammatory indices from baseline to 3 months after scaling and root planing.

Inflammatory index	Group	Baseline, median (IQR)	3 months, median (IQR)	Baseline − 3 months, median (IQR)	Within-group P value	Between-group P value at baseline	Between-group P value at 3 months	Between-group P value for change
NLR	Non-diabetic	1.90 (1.59–2.04)	1.63 (1.50–1.91)	0.24 (−0.11–0.49)	0.016	0.206	0.403	0.985
	Diabetes	2.00 (1.59–2.37)	1.69 (1.52–2.00)	0.10 (−0.17–0.69)	0.014			
SII	Non-diabetic	482.60 (368.00–521.53)	407.00 (373.50–485.41)	42.33 (−47.79–110.27)	0.043	0.237	0.171	0.401
	Diabetes	497.06 (431.69–629.60)	431.10 (384.00–500.00)	57.31 (−28.52–155.33)	0.003			

Both groups consisted of patients with periodontitis. Non-diabetic, non-diabetic periodontitis group; Diabetes, diabetes-associated periodontitis group. Data are presented as median and interquartile range because NLR and SII were not normally distributed. NLR was calculated as the absolute neutrophil count divided by the absolute lymphocyte count. SII was calculated as the platelet count multiplied by the absolute neutrophil count and divided by the absolute lymphocyte count. Within-group comparisons were performed using the Wilcoxon signed-rank test. Between-group comparisons were performed using the Mann–Whitney U test. Change was calculated as the baseline value minus the 3-month value. IQR, interquartile range; NLR, neutrophil-to-lymphocyte ratio; SII, systemic immune-inflammation index.

**Figure 4:**
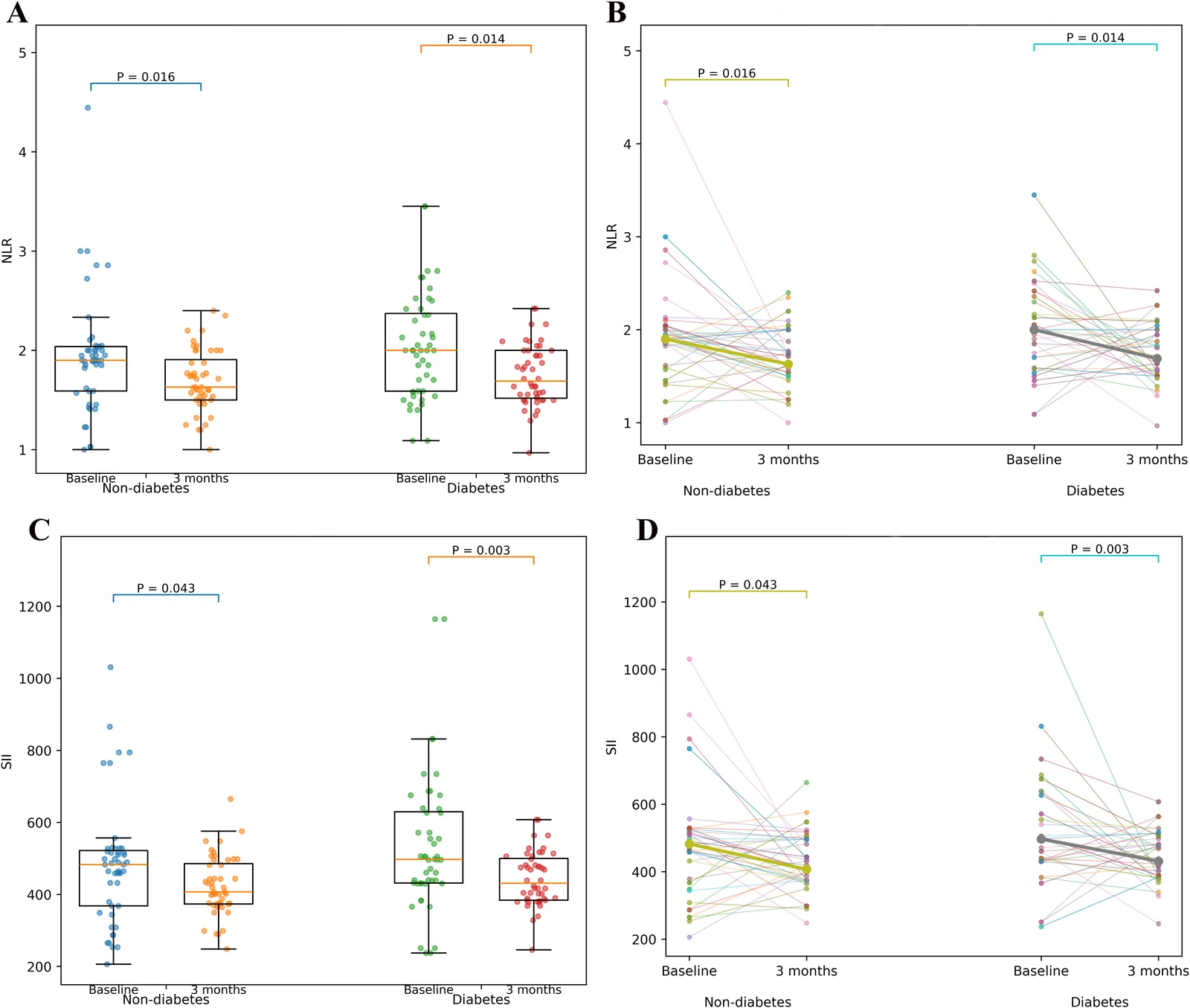
Peripheral blood inflammatory indices at baseline and 3 months after scaling and root planing. **(A)** Distribution of NLR; **(B)** individual paired changes in NLR; **(C)** distribution of SII; and **(D)** individual paired changes in SII. Box plots show medians and interquartile ranges. NLR, neutrophil-to-lymphocyte ratio; SII, systemic immune-inflammation index.

For NLR, no significant between-group differences were observed at baseline, at 3 months, or in the magnitude of change (*P* = 0.206, *P* = 0.403, and *P* = 0.985, respectively). In the non-diabetes group, median NLR decreased from 1.90 (IQR, 1.59–2.04) at baseline to 1.63 (IQR, 1.50–1.91) at 3 months (*P* = 0.016). In the diabetes group, median NLR decreased from 2.00 (IQR, 1.59–2.37) to 1.69 (IQR, 1.52–2.00) (*P* = 0.014).

For SII, no significant between-group differences were observed at baseline, at 3 months, or in the magnitude of change (*P* = 0.237, *P* = 0.171, and *P* = 0.401, respectively). In the non-diabetes group, median SII decreased from 482.60 (IQR, 368.00–521.53) at baseline to 407.00 (IQR, 373.50–485.41) at 3 months (*P* = 0.043). In the diabetes group, median SII decreased from 497.06 (IQR, 431.69–629.60) to 431.10 (IQR, 384.00–500.00) (*P* = 0.003).

Examination of the underlying blood-cell components showed that, in the non-diabetes group, the median platelet count was 252.00 (IQR, 229.50–256.75) ×10^9^/L at baseline and 248.50 (IQR, 245.75–251.50) ×10^9^/L at 3 months; the absolute lymphocyte count increased from 2.00 (IQR, 1.90–2.20) ×10^9^/L to 2.35 (IQR, 2.10–2.53) ×10^9^/L; and the absolute neutrophil count was 3.75 (IQR, 3.48–4.00) ×10^9^/L at baseline and 4.00 (IQR, 3.40–4.40) ×10^9^/L at 3 months. In the diabetes group, the corresponding values were 249.00 (IQR, 241.00–291.00) and 254.50 (IQR, 250.00–257.50) ×10^9^/L for platelet count, 2.00 (IQR, 1.90–2.20) and 2.20 (IQR, 2.08–2.40) ×10^9^/L for absolute lymphocyte count, and 3.80 (IQR, 3.30–4.60) and 3.90 (IQR, 3.60–4.40) ×10^9^/L for absolute neutrophil count, respectively. Absolute lymphocyte counts increased significantly from baseline to 3 months in both groups (both *P* < 0.001), whereas neither platelet count nor absolute neutrophil count changed significantly within either group (all *P* > 0.05). Platelet count differed between groups at 3 months (*P* < 0.001); however, there was no significant between-group difference in the magnitude of platelet-count change (*P* = 0.988). This isolated difference at the follow-up time point was therefore not interpreted as evidence of a differential longitudinal response.

Overall, NLR and SII decreased within both groups during follow-up, but no statistically significant between-group differences were detected in their baseline levels, 3-month levels, or magnitudes of change. The distributional and paired participant-level patterns underlying these results are shown in **Figure 4**.

## 4 Discussion

This prospective observational study characterized clinical and inflammatory changes following routine SRP in patients with periodontitis with and without diabetes. Improvements in periodontal clinical status and reductions in gingival crevicular fluid TNF-α were observed during the 3 months following routine SRP in both groups. The reconstructed blood-derived indices, NLR and SII, also decreased during the 3-month follow-up, although neither their absolute levels nor their magnitudes of change differed significantly according to diabetes’ status. However, participants with diabetes had higher residual BI and gingival crevicular fluid TNF-α levels at 3 months, while the within-group increase in IL-10 reached statistical significance only in the non-diabetic group. These findings are consistent with greater residual local inflammatory activity in participants with diabetes. However, interpretation of these between-group patterns requires caution because BMI distribution differed significantly at baseline, with overweight or obesity being more frequent in the diabetes-associated periodontitis group. Therefore, the observed differences cannot be attributed to diabetes status alone. Overall, the between-group differences were more apparent in local periodontal inflammatory measures, particularly BI and gingival crevicular fluid TNF-α, than in the blood-derived systemic inflammatory indices. Although NLR and SII decreased within both groups during follow-up, neither their absolute levels nor their magnitudes of change differed significantly between groups.

The present findings should be considered alongside those of Yue et al., who evaluated periodontal clinical parameters and gingival crevicular fluid TNF-α, IL-1β, and IL-10 over a similar 3-month period in patients with periodontitis with and without diabetes (18). Both studies showed substantial reductions in TNF-α after SRP and higher post-treatment TNF-α levels in participants with diabetes. However, some clinical findings differed: Yue et al. reported improvements in the principal periodontal parameters, whereas CAL did not change significantly in our cohort and post-treatment BI remained higher in the diabetes group. The present study additionally assessed platelet, neutrophil, and lymphocyte counts and the derived NLR and SII. Although NLR and SII decreased within both groups, their levels and magnitudes of change did not differ significantly according to diabetes status. Together, these findings suggest that diabetes-related differences were more apparent in residual local inflammatory activity than in the measured blood-derived inflammatory indices.

Mechanical removal of subgingival plaque biofilm and calculus is an established component of periodontal care and provides a plausible biological context for the reductions in PD and BI observed during follow-up (28, 29). CAL did not change significantly in either group during the 3-month observation period. Previous studies suggest that periodontal tissue remodeling and clinically detectable attachment changes may require longer follow-up, while irreversible periodontal tissue damage may also limit short-term changes in CAL (30, 31). The higher BI observed at 3 months in participants with diabetes occurred despite broadly similar longitudinal changes in PLI. One possible explanation, based on previous biological evidence, is that diabetes-related vascular and inflammatory alterations may contribute to persistent gingival bleeding. Chronic hyperglycemia and AGE accumulation have been associated with endothelial dysfunction and microvascular abnormalities that could plausibly influence gingival bleeding responses (32). However, vascular function, AGE accumulation, and gingival microcirculation were not directly measured in the present study; therefore, these mechanisms should be regarded as hypotheses rather than direct explanations derived from this cohort.

Because GCF samples from two periodontal sites were pooled for each participant, the cytokine values represent participant-level pooled measurements and should not be interpreted as reflecting inflammatory activity at any single periodontal site. TNF-α levels in gingival crevicular fluid were significantly lower at 3 months than at baseline in both groups. This temporal decrease is consistent with reduced local inflammatory activity during follow-up. Given that TNF-α can activate osteoclast precursors and amplify bone-resorption signaling by increasing the RANKL/OPG ratio, the lower TNF-α levels observed at 3 months may reflect a less pro-resorptive local inflammatory milieu (33, 34). Importantly, the absolute TNF-α level after treatment remained significantly higher in the diabetes group than in the non-diabetes group. This finding is closely related to the persistent induction of macrophage polarization toward the pro-inflammatory M1 phenotype under hyperglycemic conditions. By binding to its receptor RAGE, AGEs activate the NF-κB signaling pathway and maintain continuous transcription of pro-inflammatory genes in periodontal tissues, so that local inflammation resolves more slowly even after plaque control has improved (34). By contrast, IL-1β did not show a statistically significant change from baseline to 3 months in either group. Several hypotheses may be considered when interpreting this finding. Previous work suggests that the concentration of IL-1β detected in GCF may not fully represent its activity within periodontal tissues, and interindividual genetic variation may also contribute to heterogeneity in IL-1β expression and response (35, 36). However, neither tissue-bound IL-1β nor IL-1-related genetic polymorphisms were assessed in the present study. These possibilities therefore remain speculative and should not be regarded as explanations established by the current data. IL-10 increased significantly from baseline to 3 months in the non-diabetic group, whereas the within-group increase did not reach statistical significance in participants with diabetes. However, the magnitude of IL-10 change did not differ significantly between groups, and this pattern should therefore be interpreted cautiously. Previous studies have described impaired regulatory T-cell function and altered anti-inflammatory signaling under chronic hyperglycemic conditions (37, 38), which provide possible biological hypotheses for differences in inflammatory resolution. Nevertheless, Treg/Th17 balance, regulatory T-cell function, and related signaling pathways were not measured in the present study. Accordingly, the observed IL-10 pattern cannot be taken as direct evidence of altered Treg/Th17 balance or impaired immune-regulatory recovery in participants with diabetes. Taken together, these observations are consistent with impaired immune regulation in diabetes and suggest that recovery toward an anti-inflammatory profile may have been less pronounced in participants with diabetes during the 3-month follow-up.

An important consideration in interpreting these between-group findings is the baseline imbalance in BMI. Overweight or obesity was more common in the diabetes-associated periodontitis group than in the non-diabetic group (*P* = 0.003). Because adiposity is closely related to metabolic and inflammatory status, this imbalance could have contributed to differences in periodontal inflammation or inflammatory biomarker levels during follow-up. The present analyses did not specifically isolate the independent contributions of diabetes status and BMI. Therefore, the higher residual BI and TNF-α levels observed at 3 months in participants with diabetes should not be interpreted as being attributable exclusively to diabetes itself. Rather, BMI should be considered a potential confounder when interpreting these between-group differences.

In contrast to the clearer between-group differences observed in local periodontal inflammatory measures, both NLR and SII decreased within the two groups during follow-up, but no statistically significant between-group differences were detected at baseline, at 3 months, or in the magnitude of change. Absolute lymphocyte counts increased in both groups, whereas platelet and neutrophil counts did not change significantly within either group. Thus, the reduction in NLR was mainly associated with increased lymphocyte counts, while the change in SII reflected the combined variation of all three components (39). The isolated difference in platelet count at 3 months was not accompanied by a difference in platelet-count change and should therefore not be interpreted as a diabetes-specific longitudinal response. Importantly, NLR and SII are nonspecific composite indices derived from circulating blood-cell counts and may be influenced by metabolic control, glucose-lowering therapy, transient systemic inflammatory conditions, physiological variation, behavioral factors, and other systemic conditions. In the present cohort, the baseline imbalance in BMI may represent an additional source of confounding. Accordingly, the absence of statistically significant between-group differences in NLR and SII should not be interpreted as evidence that the two groups had equivalent systemic inflammatory status or that diabetes status was unrelated to systemic inflammation. Rather, the limited specificity of these indices and potential residual confounding constrain the interpretation of these null between-group findings.

In the diabetes-associated periodontitis group, HbA1c decreased numerically by 0.30 percentage points over 3 months; however, the 95% confidence interval included no change and the paired comparison was not statistically significant. The observed effect size was small. Therefore, these findings should not be interpreted as demonstrating improved glycemic control. Although systematic reviews have reported modest HbA1c reductions after periodontal therapy, the present study was not designed or powered to establish a metabolic treatment effect and did not control for changes in glucose-lowering medication, diet, physical activity, or other aspects of diabetes management (40–42). The observed reduction should therefore be regarded as exploratory and hypothesis-generating.

Several limitations should be acknowledged. First, this was a single-center prospective observational cohort rather than a randomized or investigator-assigned intervention study. Although all participants received routine SRP according to the same clinical treatment schedule, no untreated concurrent comparison group was included; therefore, the longitudinal changes observed over 3 months cannot be definitively attributed to SRP. In addition, although standardized oral hygiene instructions were provided, individual adherence was not systematically quantified, and concurrent changes in diabetes management or medication use may have influenced the findings. Second, BMI distribution differed significantly between groups at baseline, with overweight or obesity being more frequent among participants with diabetes. Because the analyses were unadjusted, the independent contributions of diabetes status and BMI cannot be separated, and the observed between-group differences should not be attributed exclusively to diabetes. Third, although the two repeated assessments were analyzed using paired tests and between-group comparisons of participant-level change values, group and time were not modeled simultaneously within a formal repeated-measures or mixed-effects framework; therefore, these comparisons should not be interpreted as formal group × time interaction tests. Fourth, the longitudinal analyses were restricted to participants who completed the 3-month follow-up, and no imputation of missing data was performed. Although attrition did not differ significantly between groups, complete-case analysis may still have introduced selection bias if follow-up completers differed systematically from those lost to follow-up. Fifth, NLR and SII are nonspecific inflammatory indices influenced by multiple systemic and metabolic factors, limiting their specificity for periodontitis-related inflammation. Sixth, GCF samples from two periodontal sites were pooled for participant-level cytokine measurement, preventing assessment of site-specific inflammatory responses and within-participant site heterogeneity; in addition, the frequency of replacement-site use was not separately tabulated. Finally, the approximately 3-month follow-up was relatively short, HbA1c was an exploratory outcome for which the study was not specifically powered, and mechanistic pathways and subgingival microbial composition were not directly assessed. These limitations support cautious, hypothesis-generating interpretation of the findings rather than causal or treatment-efficacy conclusions.

## 5 Conclusion

In this 3-month prospective observational follow-up, routine SRP was associated with lower PD, BI, and PLI and lower gingival crevicular fluid TNF-α levels in patients with periodontitis with and without diabetes, whereas CAL did not change significantly. Residual local inflammation remained greater in participants with diabetes, as reflected by higher BI and gingival crevicular fluid TNF-α levels at 3 months, although these between-group differences should be interpreted cautiously because of potential residual confounding. NLR and SII decreased within both groups, but no significant between-group differences were detected in their levels or magnitudes of change. HbA1c decreased numerically but not significantly in participants with diabetes, and this exploratory finding does not support a conclusion of improved glycemic control.

## Data Availability

The original contributions presented in the study are included in the article/**Supplementary Material**. Further inquiries can be directed to the corresponding authors.

## Supplementary material

Supplementary Figure 1 Individual paired changes in HbA1c from baseline to 3 months in participants with diabetes. Lines connect measurements from the same participant. HbA1c, glycated hemoglobin.
