# Supplementary material for: Clinical and inflammatory changes following routine scaling and root planing in periodontitis patients with and without diabetes: a prospective observational cohort study
Source: Front Endocrinol (Lausanne). 2026 Sep 24;17:1937181. doi: 10.3389/fendo.2026.1937181 (PMC13645523; doi:10.3389/fendo.2026.1937181)
Supplement: Supplementary file 1 [file SupplementaryFile1.docx]

**Instructions:** This questionnaire is designed to collect baseline demographic and health-related information. Please answer each question accurately. For questions with options, please tick (✓) the appropriate box. All information will be kept strictly confidential and used only for research purposes.

**Section A: General Information**

Field Response

A1. Participant ID ______________

A2. Date of birth (DD/MM/YYYY) ___ / ____ / ________

A3. Age (years) _____________

A4. Sex ☐ Male	☐ Female

A5. Height (cm) ______________

A6. Weight (kg) ______________

A7. Body Mass Index (BMI) ______________ kg/m² (to be calculated by investigator)

A8. BMI category (investigator-assessed) ☐ Underweight/Normal (< 25 kg/m²) ☐ Overweight/Obesity (≥ 25 kg/m²)

**Section B: Lifestyle Factors**

B1. Do you currently smoke or have you smoked in the past?

☐ No, never smoked

☐ Yes, current smoker

☐ Yes, former smoker (quit > 6 months ago)

B2. If yes to B1, how many cigarettes per day? ______________ cigarettes/day

B2a. Duration of smoking (years) ______________ years

B3. Do you consume alcohol?

☐ No, never or rarely

☐ Yes, currently

B4. If yes to B3, how many standard drinks per week? ______________ drinks/week

**Section C: Diabetes-Related Information (For participants with diabetes only)**

C1. Have you been diagnosed with diabetes mellitus? ☐ Yes	☐ No

C2. Date of diabetes diagnosis (MM/YYYY) ____ / ________

C3. Duration of diabetes (years) ______________

C4. Type of diabetes ☐ Type 1	☐ Type 2	☐ Other: ______________

C5. Current glucose-lowering treatment regimen (tick all that apply)

☐ Diet and exercise only

☐ Oral hypoglycemic agents (specify): ______________

☐ Insulin (specify type and dose): ______________

☐ GLP-1 receptor agonists

☐ Other: ______________

☐ No current treatment

C6. Most recent fasting blood glucose (mmol/L) ______________ mmol/L

C7. Most recent HbA1c (%) ______________ %

C8. Have you experienced any diabetes-related complications?

☐ No ☐ Yes: ☐ Retinopathy	☐ Nephropathy	☐ Neuropathy	☐ cardiovascular disease	☐ Other: ______________

**Section D: Medical History and Medication Use**

D1. Do you have any other active systemic inflammatory diseases?	☐ Yes	☐ No

If yes, specify: ______________

D2. Do you have any autoimmune disorders? ☐ Yes	☐ No

If yes, specify: ______________

D3. Are you currently pregnant or lactating? ☐ Yes	☐ No

D4. Have you used any of the following medications in the past 3 months? (tick all that apply) ☐ Immunosuppressants

☐ Non-steroidal anti-inflammatory drugs (NSAIDs)

☐ Glucocorticoids (systemic)

☐ None of the above

D5. Have you received systemic antibiotic therapy in the past 6 months?

☐ Yes	☐ No

D6. Have you received any professional periodontal treatment in the past 6 months?

☐ Yes	☐ No

D7. Have you undergone systemic hormone therapy, radiotherapy, or chemotherapy in the past 6 months?

☐ Yes	☐ No

**Section E: Oral Health Examination Information**

E1. Total number of remaining natural teeth ______________

E2. Number of teeth in each quadrant Upper right: ____	Upper left: ____

Lower right: ____	Lower left: ___

E3. Do any teeth have obvious hard tissue defects, wedge-shaped defects, furcation involvement, or extensive restorations?

☐ Yes	☐ No

If yes, specify: _______________

**Section F: Confirmation of Eligibility (Investigator use)**

F1. Age between 18 and 65 years ☐ Yes	☐ No

F2. At least 20 natural teeth remaining ☐ Yes	☐ No

F3. No fewer than 5 teeth in each quadrant ☐ Yes	☐ No

F4. No systemic antibiotic therapy within 6 months ☐ Yes	☐ No

F5. No professional periodontal treatment within 6 months ☐ Yes	☐ No

F6. Meets all inclusion criteria and none of the exclusion criteria ☐ Yes	☐ No

**Section G: Informed Consent**

G1. Has the participant been fully informed of the study purpose and procedures?

☐ Yes	☐ No

G2. Has written informed consent been obtained? ☐ Yes	☐ No

G3. Participant signature ______________________________

G4. Date (DD/MM/YYYY) ___ / ____ / ________

G5. Investigator signature	______________________________

G6. Date (DD/MM/YYYY) ___ / ____ / ________

**End of Questionnaire**
