# Supplementary material for: Clinical and inflammatory changes following routine scaling and root planing in periodontitis patients with and without diabetes: a prospective observational cohort study
Source: Front Endocrinol (Lausanne). 2026 Sep 24;17:1937181. doi: 10.3389/fendo.2026.1937181 (PMC13645523; doi:10.3389/fendo.2026.1937181)
Supplement: Supplementary file 2 [file SupplementaryFile2.docx]

**Supplementary Table S1.** Platelet, absolute neutrophil, and absolute lymphocyte counts used to calculate NLR and SII at baseline and 3 months.

| **Blood-cell parameter** | **Group** | **Baseline, median (IQR)** | **3 months, median (IQR)** | **Baseline − 3 months, median (IQR)** | **Within-group *P* value** | **Between-group *P* value at baseline** | **Between-group *P* value at 3 months** | **Between-group *P* value for change** |
| --- | --- | --- | --- | --- | --- | --- | --- | --- |
| Platelet count (×10⁹/L) | Non-diabetes | 252.0 (229.5–256.8) | 248.5 (245.8–251.5) | 1.5 (-17.2–11.5) | 0.809 | 0.507 | 0.000 | 0.988 |
| Platelet count (×10⁹/L) | Diabetes | 249.0 (241.0–291.0) | 254.5 (250.0–257.5) | -4.5 (-19.0–41.5) | 0.922 |  |  |  |
| Absolute lymphocyte count (×10⁹/L) | Non-diabetes | 2.00 (1.90–2.20) | 2.35 (2.10–2.52) | -0.25 (-0.60–-0.10) | 0.000 | 0.944 | 0.123 | 0.806 |
| Absolute lymphocyte count (×10⁹/L) | Diabetes | 2.00 (1.90–2.20) | 2.20 (2.08–2.40) | -0.25 (-0.60–0.10) | 0.000 |  |  |  |
| Absolute neutrophil count (×10⁹/L) | Non-diabetes | 3.75 (3.48–4.00) | 4.00 (3.40–4.40) | -0.20 (-0.63–0.50) | 0.391 | 0.532 | 0.678 | 0.649 |
| Absolute neutrophil count (×10⁹/L) | Diabetes | 3.80 (3.30–4.60) | 3.90 (3.60–4.40) | 0.05 (-0.50–0.63) | 0.992 |  |  |  |

*Data are presented as median (interquartile range) because the distributions of the blood-cell variables were non-normal in at least one study group or time point. Within-group comparisons were performed using the Wilcoxon signed-rank test, and between-group comparisons were performed using the Mann–Whitney U test. Change was calculated as the baseline value minus the 3-month value. NLR was calculated as the absolute neutrophil count divided by the absolute lymphocyte count, and SII was calculated as the platelet count multiplied by the absolute neutrophil count and divided by the absolute lymphocyte count. IQR, interquartile range; NLR, neutrophil-to-lymphocyte ratio; SII, systemic immune-inflammation index.*
